# Feasibility of Locating Leakages in Sewage Pressure Pipes Using the Distributed Temperature Sensing Technology

**DOI:** 10.1007/s11270-017-3250-7

**Published:** 2017-02-01

**Authors:** Benjamin Apperl, Alexander Pressl, Karsten Schulz

**Affiliations:** 1 Institute of Water Management, Hydrology and Hydraulic Engineering (IWHW), University of Natural Resources and Life Sciences (BOKU), Muthgasse 18, Vienna, Austria; 2 Institute of Sanitary Engineering and Water Pollution Control (SIG), University of Natural Resources and Life Sciences (BOKU), Muthgasse 18, Vienna, Austria

**Keywords:** Pipe leakage detection, Distributed temperature sensing, Pressure pipes, Feasibility study, Wastewater

## Abstract

The cost effective maintenance of underwater pressure pipes for sewage disposal in Austria requires the detection and localization of leakages. Extrusion of wastewater in lakes can heavily influence the water and bathing quality of surrounding waters. The Distributed Temperature Sensing (DTS) technology is a widely used technique for oil and gas pipeline leakage detection. While in pipeline leakage detection, fiber optic cables are installed permanently at the outside or within the protective sheathing of the pipe; this paper aims at testing the feasibility of detecting leakages with temporary introduced fiber optic cable inside the pipe. The detection and localization were tested in a laboratory experiment. The intrusion of water from leakages into the pipe, producing a local temperature drop, served as indicator for leakages. Measurements were taken under varying measurement conditions, including the number of leakages as well as the positioning of the fiber optic cable. Experiments showed that leakages could be detected accurately with the proposed methodology, when measuring resolution, temperature gradient and measurement time were properly selected. Despite the successful application of DTS for leakage detection in this lab environment, challenges in real system applications may arise from temperature gradients within the pipe system over longer distances and the placement of the cable into the real pipe system.

## Introduction

The organization of a comprehensive wastewater treatment network in Austria required the construction of centralized wastewater treatment plants, as well as supply pipes, transporting the sewage from the polluter to the treatment plant. Given economic constraints, the supply pipe system was planned along the shortest, technically feasible distances. Consequently, in the 1970s and 1980s, pressure pipes for sewage transport have been installed in several scenic lakes in Austria (Pressl et al. 2015). Today, about 160 km of wastewater pressure lines are placed at the bottom of Austrian lakes.

The technical lifetime of these pressure pipes was expected in the range of 50 years. Pressl et al. (2015) report only 15 damages in Austria (mainly pipe cracks), having an effect on the continuous wastewater disposal. Almost all pipe cracks were localized in the shallow areas of the lakes. Deeper pipe sections were so far not affected by cracks but can be damaged through small leakages. Extrusion of sewage into the lake system through leakages has the potential to strongly deteriorate the lake water quality and thereby the ecological system. Negative impacts on water quality might also provoke health risks, especially during the bathing season, causing also economic losses due to reduced tourism in the region. The European directive of bathing water quality (2006/7/EC 2006) pushes the member states to implement adequate management measures to protect the environment and public health by reducing the lake water pollution and to protect it from further deterioration. Given the advanced age and potential for leakages of the used lake-pressure pipe system, a feasible technology for an economic and efficient repair set of even small leakages is urgently required.

The current state-of-the-art in sewage pipe inspection consists of several monitoring methods (Duran et al. 2002; Liu and Kleiner 2013; Steel and McGhee 1991). Limited by the pipe material and the surrounding environment (buried or not buried), the following procedures are mainly used for inspection of sewage pressure pipes: (i) pump data analysis, (ii) optical inspection (Duran et al. 2002), and (iii) static pressure test. Other used procedures are based on continuous measurements, as (iv) pipe pressure (Dohmann et al. 1999) and (v) flow measurements (Rutsch et al. 2008). Methods (i)–(iii) are conducted periodically. All methods give reliable information about the existence of leakages; however, only the tethered optical inspection allows the location of the leakages along the pipe. The optical inspection, based on closed-circuit television systems, has a relatively poor performance (Duran et al. 2002) and has also the disadvantage of being time consuming and expensive, when pipes exceed a certain length (100 m and more) and are placed in deep water. A further common disadvantage of these methods is that small leakages are often overlooked (Zhang 1996), or they suffer from a restricted operational range (Colombo et al. 2009).

The Distributed Temperature Sensing (DTS) technology provides a mean to circumvent the difficulties and limitations. DTS systems allow to detect and locate temperature changes along a fiber optic cable up to a length of 10 to 30 km in a very high spatial and temporal resolution (Apperl et al. 2015; Selker et al. 2006; Smolen and Spek 2003). DTS has already been used in storm sewers to detect illicit connections. Since 20 years, DTS is a widely used technology used in pipeline and process engineering (Meulman et al. 2013; Nikles et al. 2002, 2016). DTS is classified as an external-based system, measuring the temperature around the pipeline with a permanently installed fiber optics (FO) cables near the pipe (Frings 2011). Local leakages produce measureable temperature anomalies in the vicinity. Depending on the substance transported in the pipe, a local temperature drop or temperature increase is observable. Oil is heated for transport; consequently, a leakage produces a local warming. Gas is transported under high pressure and produces a local temperature drop due to the Joule Thomson effect (Nikles et al. 2002). The current detection limits are in the order of 0.01% of the total throughput for oil leaks (Nikles et al. 2016). The FO sensor cables are placed permanently and are either installed exclusively for pipeline monitoring or existing telecommunication. FO cables are used as they are placed normally in the vicinity of pipelines.

Unlike the typical permanent placement of the FO cables, this paper aims at testing a methodology for sewage pipe inspection without cost-intensive permanent placement of FO cables but with the advantages of accurate spatial detection of leakages of DTS. Furthermore, it should be rapidly installed and cost-efficient. The idea of the inspection system for sewage pipe leakage detection is to measure temperature changes with the DTS cable installed temporarily within the pipe. The temperature gradient between the water outside (lake hypolimnion) and inside the pipe might be generated by filling the pipe system with warmer surface water in the summer months from the warmer epilimnion. The application of negative pressure within the pipe system will cause the intrusion of cold hypolimnion water into the pipe system. The penetrating colder water will alter the local water temperature in the pipe, which will be detected and monitored by the DTS system. After finishing the tests, the cable can be removed completely.

In order to test this new monitoring concept, a medium-scale laboratory experiment was designed to test the feasibility of the method. Special focus was paid on the varying cable positioning inside the pipe as well as the limits and challenges of this methodology in the practical implementation. In the following chapter, the methodology and materials used for the experiment will be explained. An overview about the DTS technology, the approach of leakage detection via DTS in nature, and the transformation of the setup to the experimental design will be given. In section 3 and 4, the test data are presented, interpretations of the measured results are given, and the potential and difficulties for the implementation in nature are discussed.

## Materials and Methodology

### DTS Technology

The DTS technology provides temperature measurements with high temporal and spatial resolution along a FO cable (Selker et al. 2006). The DTS device is connected with at least one end of the fiber (Hausner et al. 2011). A laser pulse is emitted by the device and propagates through the FO cable, which serves as a linear sensor. A spectrometer measures the backscattered photons. By measuring the travel time, the location of backscattering in the cable can be determined (Smolen and Spek 2003). Besides the elastic scattering, the inelastic scattering, more precisely Raman and Brilluion scattering, produces shifts in the wavelength spectrum (Selker et al. 2006; Suárez et al. 2009). Raman scattering, which is used to determine the temperature in this experiment, produces wavelength shift towards higher frequencies (the anti-Stokes component) but also towards lower frequencies (Stokes component). While the magnitude of the Stokes component is temperature independent, the anti-Stokes component magnitude increases exponentially with temperature. The temperature can be inferred from the ratio of the magnitude of these two components (Ferraro et al. 2003; Selker et al. 2006; Tyler et al. 2009). The accuracy of the temperature measurements depends on the photons counted to calculate the Stokes/anti-stokes ratio. Consequently, it is directly dependent on the temporal and spatial resolution of the measurement (Ciocca et al. 2012). In the experiments, a Silixa XT-DTS^TM^ device with a maximum spatial resolution of 0.25 m and a temporal resolution of 10 s and a Brusens® temperature FO cable (Brugg Kabel AG, Brugg, Switzerland) was used.

### Measurement Approach

**Fig. 1:**
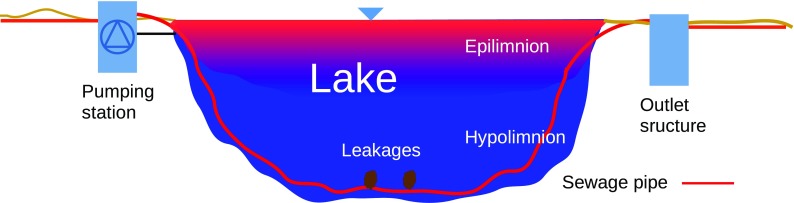
Delineation of a sewage pressure pipe through a lake

The approach for the implementation of DTS in nature is as follows: first, the FO cable has to be introduced in the pressure pipe at the pumping station (Fig. 1). To be able to introduce the FO cable, it has to be floated into position with support of the pump using a small threading device. To minimize tensile stress by friction, the specific weight of the cable should be similar to those of water. Under normal conditions, the pumping station is operated discontinuously (usually batchwise), depending on the amount of wastewater entering the station. Therefore, the inner water temperature of the pipe is mainly influenced by the lake temperature. During the stratification phase of the lake in summer, the temperature of the superficial layer is respectively higher than in the deeper layers (Dokulil 2001). The warm superficial layer, the epilimnion, is separated from the deeper, cold layer in the hypolimnion by a transition layer with sharp temperature gradient. The warmer water from the epilimnion can be pumped through the pipe when introducing the fiber optic cable, creating an artificial temperature gradient. Afterwards, the penetration of negative colder lake water into the pipe through the leakages can be provoked by, firstly, closing valves at the outlet structure and the pumping station and, secondly, setting a slightly negative pressure (about 0.1 bar) in the pipe through the installed pumps. The resulting local temperature drop at the leakages should be measurable and its location be identified.

### Experimental Design

**Fig. 2:**
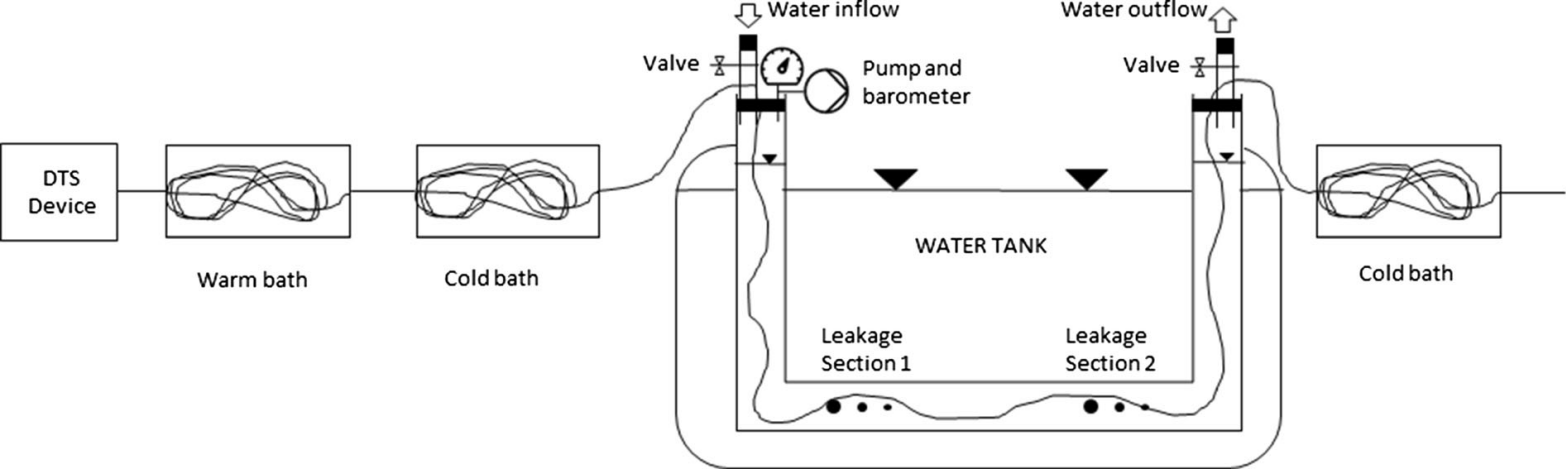
Scheme of the experimental medium-scale design

**Fig. 3:**
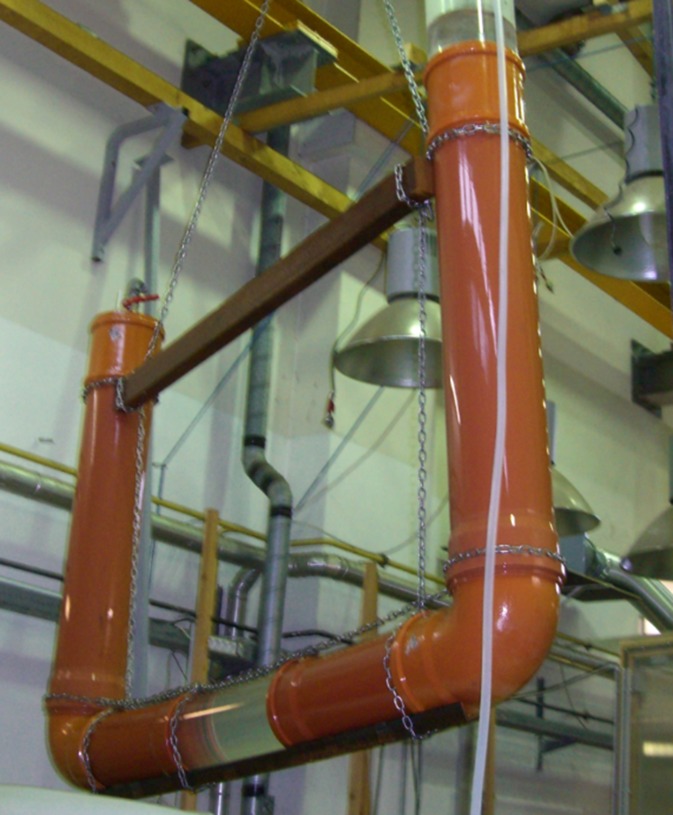
Experimental design. Six-meter-long U-shaped pipe which gets submerged in a water bath

A laboratory experiment was conducted to test the feasibility of leakage detection with an aleatory cable positioning (respectively to the pipe profile) inside the pipe, simulating conditions for leakage detection in a natural environment. A schematic of the experimental design is illustrated in Fig. 2. A 6 m long U-shaped polyvinyl chloride (PVC) pipe with a diameter of 20 cm served as a pressure pipe (Fig. 3). Idealized circular leakages were drilled with radius of 4, 6, and 8 mm at two defined sections. The DTS cable was threaded into the pipe with a taut wire. The cable positioning was located every 25 cm. At both ends, the cable passed a calibration section with an ice and a warm bath. A single-ended installation with only one connection to the instrument was chosen (Hausner et al. 2011). The calibration parameters were calculated explicitly from a set of three reference sections. The pipe was submerged almost completely in a water tank, which was filled with cold water similar to water temperatures in the hypolimnion layer. After starting the measurements, the pipe got flushed with water up to 10 °C warmer than the water in the tank and temperature measurements were taken continuously along the pipe. The simulated temperature differences originate from typical seasonal temperature differences between the epilimnion and hypolimnion (Hostetler 1995).

**Fig. 4:**
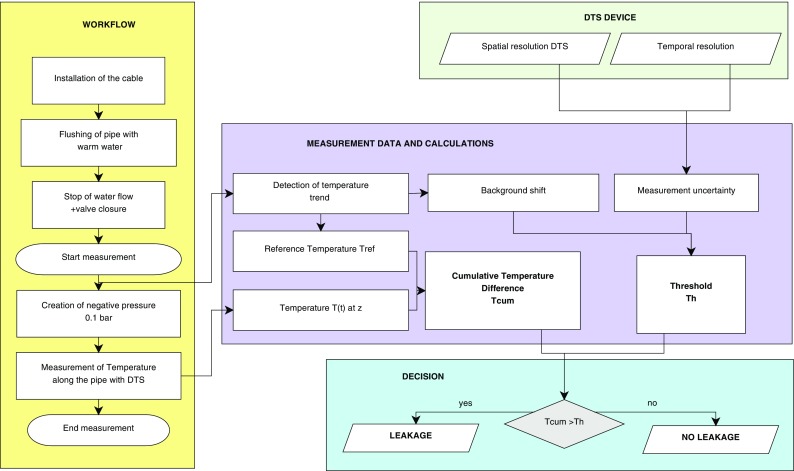
Overview of experiment workflow and data analysis. *yellow*: measuring steps; *green*: DTS adjustments; *purple*: data processing; *blue*: leakage decision criterion

Under normal pumping conditions, a water extrusion into the tank water from the pipe through a leakage is to be expected; no temperature differences would be detectable inside the pipe. To generate an inversion of the flow, firstly, the flush with warm water was stopped and the valves at the beginning and the end of the pipe were closed. After a couple of minutes, an approximated uniform temperature difference of the water inside the pipe and the water in the tank was observable. An inverse flow was induced by implementing a slightly negative pressure of 0.1 bar at the end of the pipe, provoking an intrusion of colder water from the tank into the pipe. Cold water mixed with the warmer water leading to a local temperature decrease near the leakage. An overview of the workflow can be found in Fig. 4.

### Measurements Sequences

A set of 13 measurement sequences were conducted. They differed in the following:

- Temporal resolution
- Spatial resolution
- Cable positioning
- Number and size of leakages

The temporal and spatial resolution of the DTS measurements has a strong influence on the detected temperature differences as well as on the measurement uncertainties. Lower spatial and temporal resolution might lead to buffered detected temperature differences, as well as lower measurement uncertainties. Cable positioning might be affecting the measured temperature, as sections near the pipe wall might be influenced by thermal exchange between outside and inside temperature. This would result in lower temperature measurements near the wall. The influence of spatial and temporal resolution on the localization was tested by realizing different measurement runs under identical experiment conditions. To test the wall influence on the leakage detection, another measurement series was realized with a free-floating cable and other measurement series bonding the cable at the pipe internal wall. Latter have been compared with measurements, separating the cable at least 3 cm from the wall with braces made by foamed polystyrene. Finally, tests were conducted with multiple leakages of different sizes and different spatial resolutions.

### Data Interpretation

**Fig. 5:**
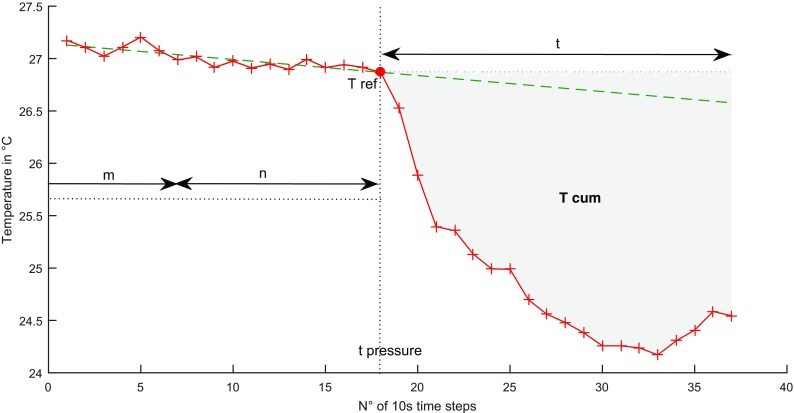
Exemplary temperature plot at a location *z* with the measured temperature (*red line*), background temperature shift (*green-dashed line*) and the temperature reference *T*_ref_ (*red point*) measured at time *t*_pressure_ = time of setting negative pressure

Temperature measurements are taken, varying the spatial resolution (sr) (0.25, 0.5, 1, and 2 m) as well as the temporal resolution (tr) (10, 30 and 60 s). The spatial resolution refers to the spatial integration scales over which a single temperature value is reported, the temporal resolution refers to integration time, and the fiber temperatures are resolved (Tyler et al. 2009). The detection of anomalies by leakages requires the detection of dissimilarities and a threshold definition (Khan et al. 2010). First are interpreted using the cumulative temperature changes *T*_cum_ (°C) during the integration time (*t*), similar as described in Sayde et al. (2010) at every measurement point z (m) along the cable (see Fig. 5):

1 $$ {T}_{\mathrm{cum}(z)}={\displaystyle \sum_{k=0}^t}\left({T}_{\mathrm{ref}(z)}-{T}_{\mathrm{ref}+ k\ (z)}\right) $$

with *T*_ref_ (°C) a reference temperature calculated from the measurements taken just before provoking the water intrusion into the pipe and *T*_ref+*k*_ the temperature measured *k* time steps after provoking the intrusion of water. Using the cumulative temperature *T*_cum_ reduces the probability of erroneous detected temperature changes caused by measurement uncertainties or erroneous measurements.

### Threshold Definition

Whether a cumulative temperature change arises from a leakage or from random noise of the measurements is determined by a predefined threshold *T*_h_, a threshold exceedance of *T*_cum_ (|*T*_cum_| > |*T*_h_|) at location *z* indicates a leakage because the probability that the temperature difference arise from uncertainties gets insignificantly small. *T*_h(*z*)_ is defined as

2 $$ {T}_{h(z)}=3\  x\ {\sigma}_{T\mathrm{cum}\left(\mathrm{sr},\mathrm{tr}\right)} + {\displaystyle \sum_{k=0}^t}\varDelta {T}_{\mathrm{background}(z)} $$

with σ_Tcum_ the standard deviation of *T*_cum_ (°C) provoked by measurement uncertainties, Δ*T*_background_ (°C) the background temperature shift occurring through thermal conduction (between outside and inner pipe water), and *t* the number of measurements for the determination of *T*_cum_. σ_Tcum_ is calculated applying a Gaussian error propagation under the assumption of normal distribution of the errors (Rice 2007). Δ*T*_background_ has been determined under static flow conditions of the system between stopping the flush and setting the negative pressure (*t*_pressure_). During this period, the only temperature changes are caused by thermal conductivity. The slope of a simple linear regression of the temperature over time in this period has been used as Δ*T*_background_ (°C/*n*).

### Determination of T_ref_

The accurate determination of the reference temperature *T*_ref_ plays a crucial role when applying the presented methodology. *T*_ref_ heavily influences the outcome of *T*_cum_ as it appears in every addend of the sum. Different methods for the determination of *T*_ref_ were tested. To reduce the uncertainty of *T*_ref_, a set of *n* measurements are used for the determination of *T*_ref_ instead of one single measurement, reducing the uncertainty (JCGM 2008):

3 $$ {\sigma}_{T\mathrm{ref}(z)}=\frac{\sigma_{T(z)}}{\sqrt{n}} $$

These measurements are taken just before initializing the negative pressure, identical to the determination of Δ*T*_background_. The derivation of *T*_ref_ was tested by calculating the arithmetic mean as well as the resulting temperature from a trend elimination by a simple linear regression just before the start of the pressure reduction. The influence of the variation of the number of measurements (*n*) and the starting point in (*m*), when the first value of *n* is taken (see Fig. 5) is examined by calculating an ensemble of *T*_ref_(*z*) with varying *m* and *n* and analyzing the consequences on *T*_h_ − *T*_cum_. An ensemble of (*m* × *n*)/2 calculations of *T*_ref_ has been conducted at every location *z* along the cable. Here,

4 $$ n+ m = {t}_{\mathrm{pressure}}={n}_{\max }-1={m}_{\max }-1 $$

with *n*_max_ the maximum number of temperature measurements available before the negative pressure is set, *m*_max_ the latest moment for taking a reference temperature measurement before the negative pressure is set at time *t*_pressure_.

## Results

### Leakage Detection

**Fig. 6:**
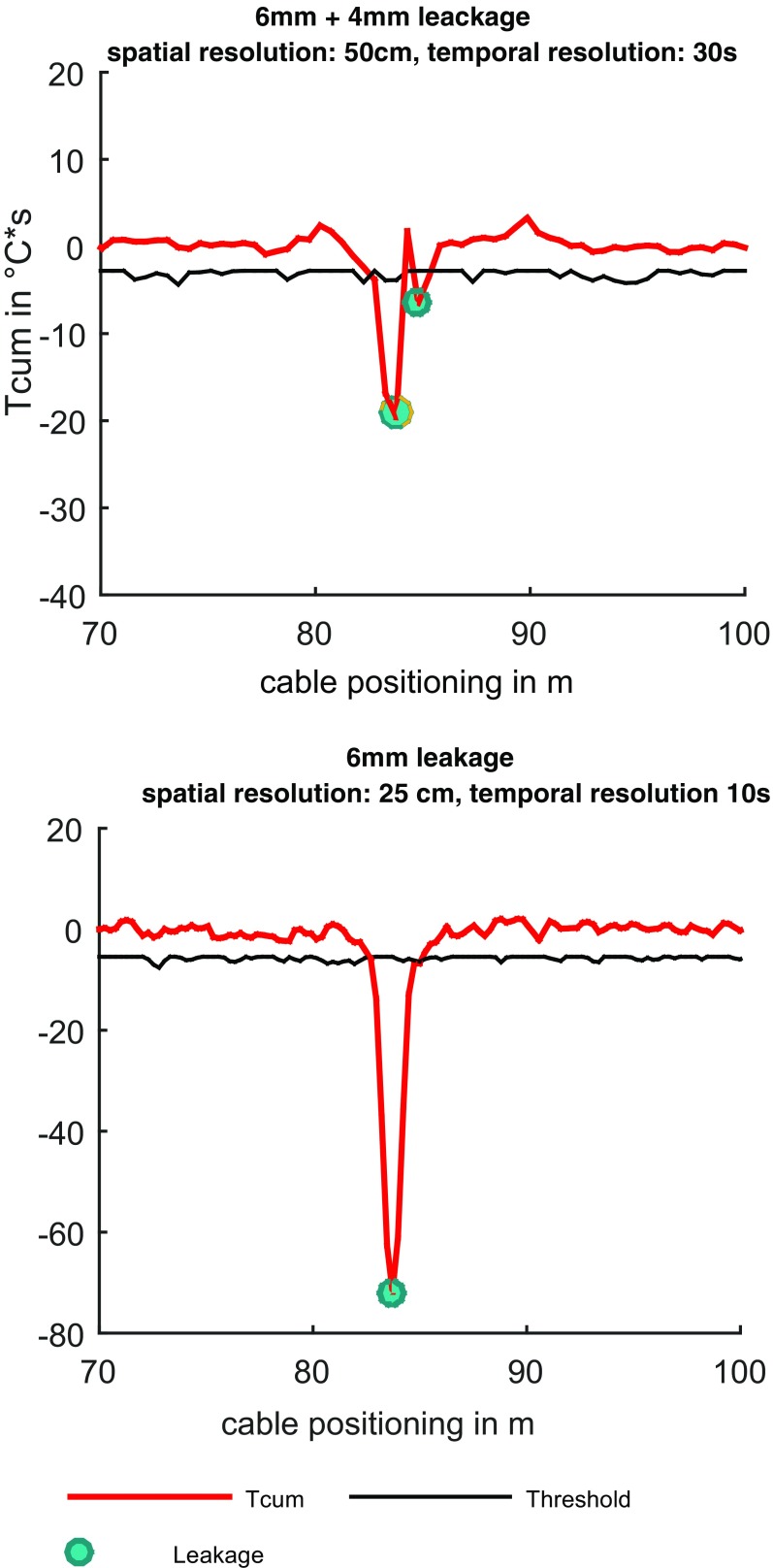
Cumulative temperature and detected leakages (*left*: two leakages; *right*: one leakage)

The analysis of *T*_cum(*z*)_ along the DTS cable showed characteristic peaks at leakages. Results show that already small leakages (4 mm) can be detected (Fig. 6). The amplitude varies according to the integration time *t*, the temperature difference between the outside and inside fluid, and the number of leakages. A series of leakages led to a drop of the negative pressure, increasing from the nearest to farthermost positioning, according to the suction valve. The number of detectable leakages corresponds with the intensity of the negative pressure and the leakage size, which in turn is limited by the mechanical stability of the tube wall. These effects have been overcome by longer integration times. Threshold *T*_h_ mainly depends on the measurement uncertainty σ_Tcum(sr,tr)_. Background temperature shifts are marginal as the measurement time is maximal *t*_max_ = 240 s and no relevant temperature changes through thermal convection are observable (−1.45 − 0 °C/min) in this time lag.

**Fig. 7:**
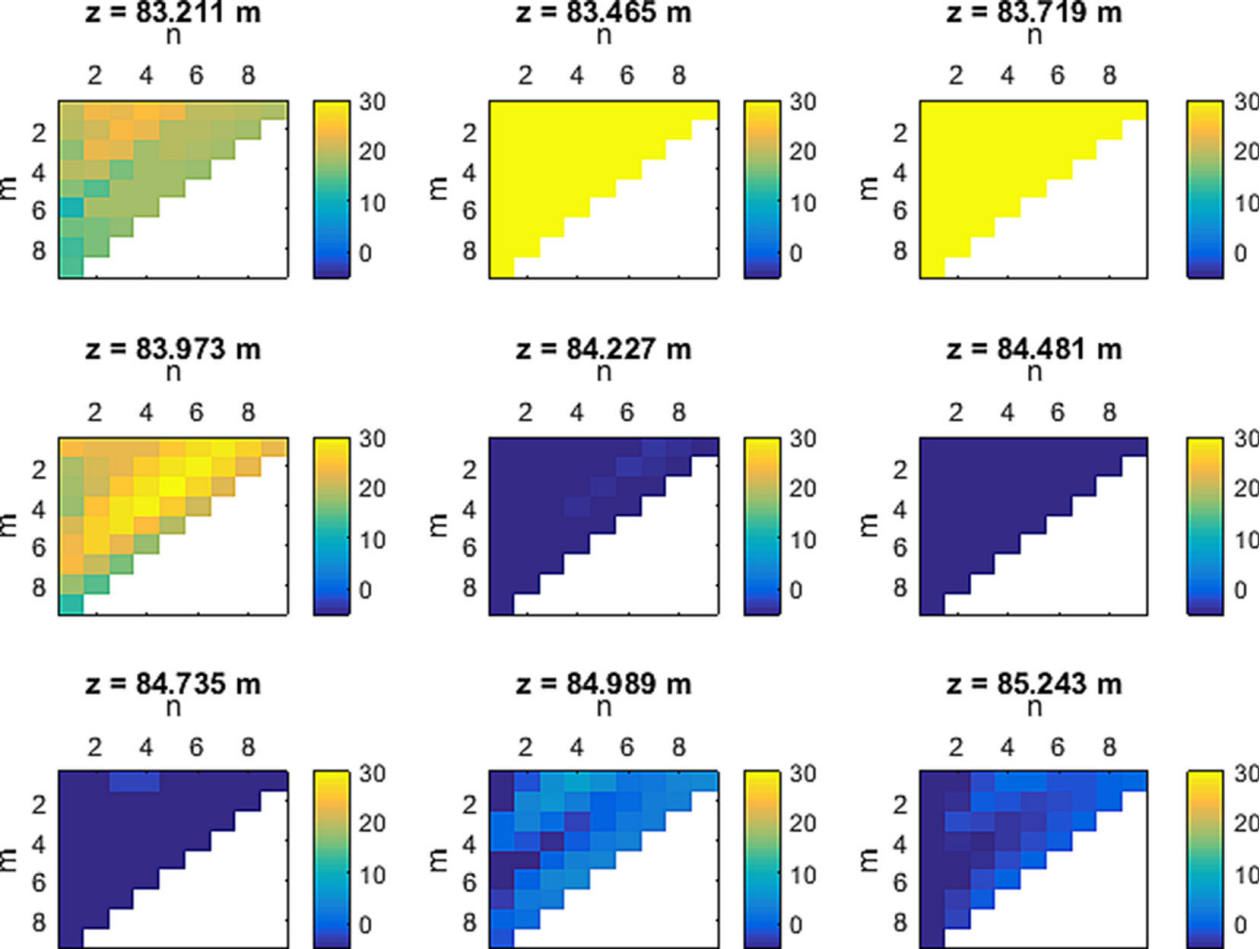
*T*_cum_ − *T*_h_ by varying parameters *n* and *m* (*n* = number of values used for determining *T*_ref_, *m* = starting point in time when first value of *n* is taken) at single locations *z*; positive values = leakage; Zero values = no leakage

For the reference temperature at every location *z* along the cable, a set of temperature values in time was used varying *m* and *n*. In Fig. 7, the influence of *m* and *n* variation on *T*_cum_ − *T*_h_ is shown. The integration time of this measurement was 200 s with a temporal resolution of 10 s and a spatial resolution of 25 cm. Trend elimination was done by linear regression just before the start of the pressure reduction. The trend elimination diminishes the sensitivity of choosing the appropriate *m* and *n* heavily, in contrast to the calculation of the arithmetic mean of the *n* values (figure not shown). Latter shows higher inconsistency in leakage identification for a location *z*.

**Fig. 8:**
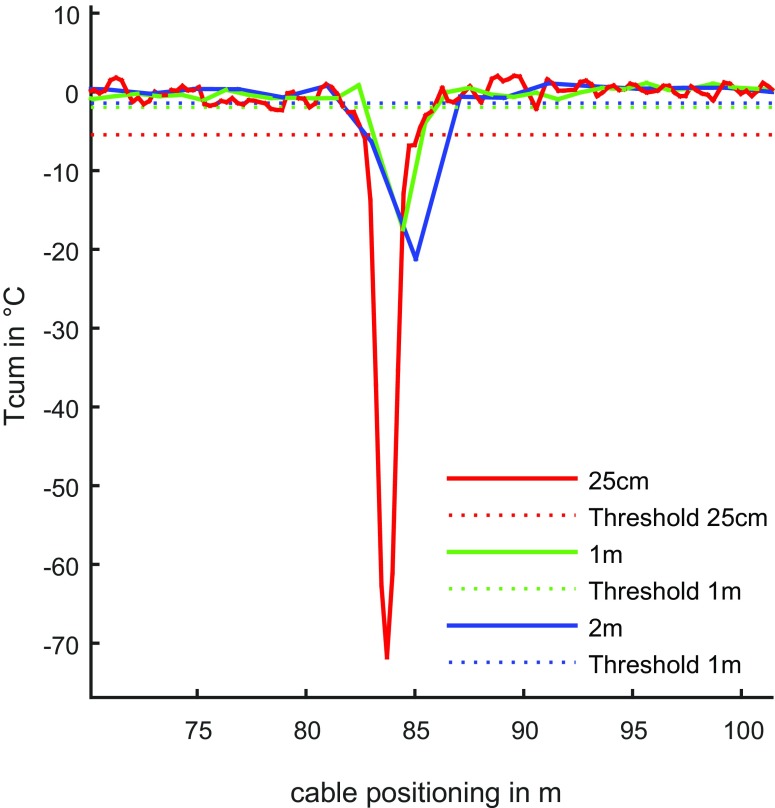
Influence of spatial resolution on the cumulative temperature *T*_cum_ and threshold *T*_h_ for a 6-mm hole at 84.5 m cable positioning (integration time = 200 s)

The effect of the spatial resolution on leakage positioning has effects on both, *T*_cum_ as well as on the threshold *T*_h._ The former diminishes at lower resolution. This is obvious, as the intruding water at the leakages is less in relation to the measurement volume at lower spatial resolution. The latter diminishes at lower spatial resolution as well because of reduced measurement uncertainty. In Fig. 8, the influence on *T*_cum_ and *T*_h_ is shown. Regarding the detection of the leakage, both, the high as well as the low spatial resolution is capable to detect leakages. The effect of lower *T*_cum_ is compensated by the lower measurement uncertainty and consequently by the lower threshold *T*_h_. Regarding the positioning of the leakage, high spatial resolution is preferable.

**Fig. 9:**
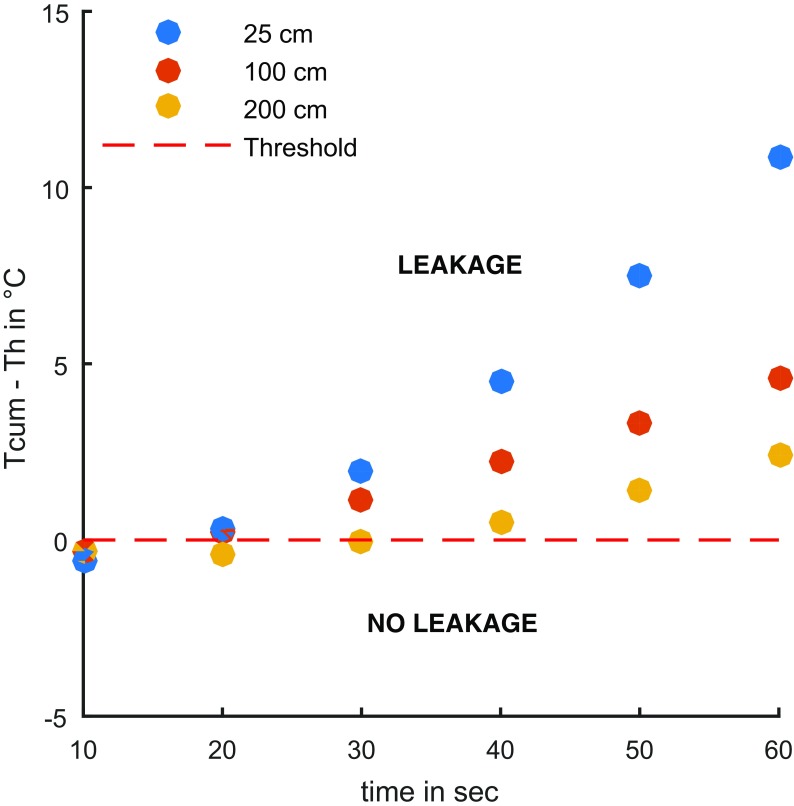
Duration of threshold exceedance at a leakage point for different spatial resolutions for a measurement interval of 10 s

The number of measurements used for the calculation of *T*_cum_ and *T*_h_ influences on the accurate leakage detection, as well as the spatial and temporal resolution on the ability for detecting leakages. A higher number of measurements increase the sample size and reduce the effects of outliers. The spatial resolution influences on the relation *T*_cum_ to *T*_h_, especially at the beginning of the measurement when water intrusion starts and the mixing volume is still low. At lower spatial resolutions, the measurement volume of a measurement is higher than at higher resolutions. Consequently, the temperature drop from water intrusion is smaller than at higher resolution. At the beginning of the measurement, the mixing volume is still small and the temperature fluctuation is within the threshold. These effects are demonstrated in Fig. 9. *T*_cum_ increased non-linearly with time after setting the negative pressure. The threshold *T*_h_ was higher at high spatial resolutions, influencing the result especially at the beginning of the measurement. Nevertheless, the leakage was detected correctly independently of the spatial resolution, if the integration time was sufficiently long. High spatial resolutions are preferable, as the influence on the measured temperature difference is more striking than the effects of higher uncertainty on *T*_h_. The integration time should be as long as possible. In the experiments, 60 to 180 s were considered optimal. The maximum is limited by the ability to hold the negative pressure in the system and by heat conduction from the outside into the pipe system. Lower spatial resolution requires longer integration times.

### Cable Positioning

**Fig. 10:**
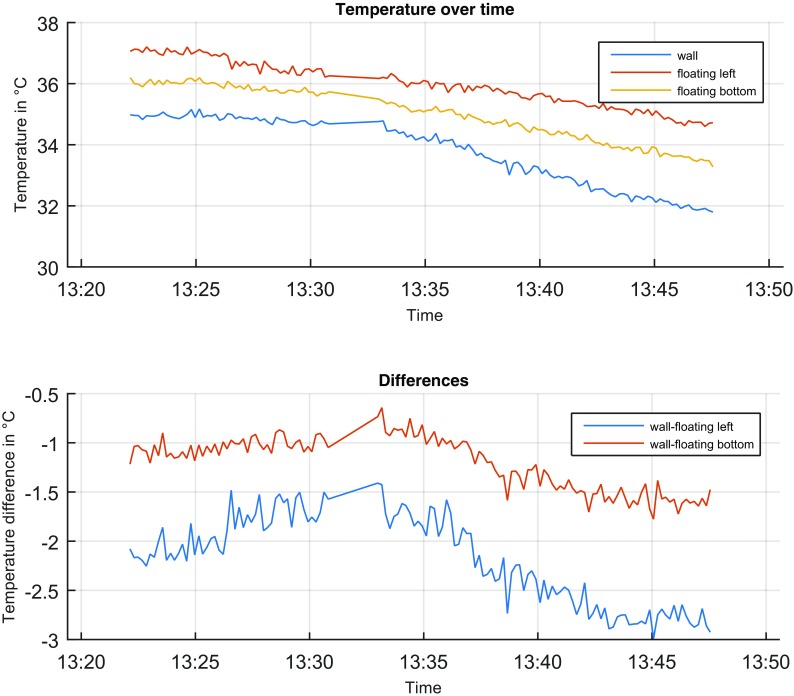
Effects of cable positioning inside the pipe (temperature of flushed water 35–37 °C). *Upper*: measured temperature values; *Lower*: temperature difference of bounded to free-floating section

The influence of the cable position was tested by either fixing sections of the cable to the inside wall with duct tape or separating it by at least 3 cm with spacers made of foamed polystyrene. A reduced measured temperature, when the cable gets in contact with the wall, was not necessarily an impediment for leakage detection. A crucial step was the accurate determination of the reference temperature *T*_ref_ along the cable. Thus, small scale variations due to the unknown relative cable positioning against the pipe wall are captured and eliminated in the calculations of *T*_cum_. However, if the cooling effect of the wall effects superimposes the effect of the temperature rise of the flushed water, the local temperature gradient between outside water temperature and the measured inside temperature might vanish or get lower than the measurement uncertainty itself. In Fig. 10, the temperature in time for both sections is shown. The temperature of the flushed water varied from 37 °C at the beginning to 35 °C at the end of the measurement (red line). The temperature in the water tank was 30 °C. The measured temperature at the wall section was continuously lower than the temperature of the flushed water with differences up to 3 °C. While this temperature difference Δ*T* is bigger than the threshold *T*_h_ for a single measurement, leakage detection is possible but might need an increased number of measurements for *T*_cum_. Since this temperature difference can change at every location *z* along the cable, a revision of the temperature along the pipe has to be realized in advance.

## Conclusions

In a laboratory experiment, the potential of an adapted methodology of the distributed temperature sensing technology for detecting leakages in pressure pipes was tested. In comparison to traditional methods, the proposed methodology is advantageous because even small leakages can be located accurately. Furthermore, the installation of the cables in existing pipes is temporary without installation of any additional technical equipment, except the DTS itself. The temperature gradient between the pipe system and the surrounding lake water can be generated by flushing superficial lake water from the epilimnion in the summer months without any cost-intensive heating of the water.

Testing several experimental designs, best results in the laboratory experiment were obtained with high spatial resolution to not overlook small leakages and short measurement intervals of 10 s. Concerning the data post-processing, the accurate determination of a reference temperature from measured DTS signals was the most crucial part. It also showed to be essential to maintain a stable temperature gradient within the pipe system. This was best achieved in the lab experiment by flushing the pipe as long as possible, before the start of the measurements. To reduce effects of uncertainty, the integration time should be chosen as long as possible in dependence of the maximum time negative pressure is uncritical for the statics of the pipe.

Further challenges may arise in the practical implementation. While a suitable measurement design in terms of spatial resolution, temporal resolution and integration time of the measurement are already conditioned by the DTS technology/system itself, it has to be designed under strong consideration of the pipe length and the effective temperature gradient along the pipe system. Also, the insertion method of the DTS cable into the pipe should be tested in consideration of the emerging tensile stress through wall frictions. The effects of pressure drops along the pipe have to be evaluated. If multiple leakages lead to a significant pressure drop, an analysis and repair in sections, starting at the closest section to the pump, could be tested.

While in the laboratory, a stable temperature gradient could be generated; in nature, much more heterogeneous temperature conditions in the pipe are expected. As a next step, we suggest to test the developed methodology under natural real system conditions, in order to explore the practical feasibility of our method for large-scale application. Overcoming the difficulties and exploiting the natural temperature gradient and the existing infrastructure, the methodology might provide a cost-attractive alternative to traditional methods in sewage pipe inspection.
